# Association between intimate partner violence and poor child growth: results from 42 demographic and health surveys

**DOI:** 10.2471/BLT.15.152462

**Published:** 2016-05-02

**Authors:** Jeanne Chai, Günther Fink, Sylvia Kaaya, Goodarz Danaei, Wafaie Fawzi, Majid Ezzati, Jeffrey Lienert, Mary C Smith Fawzi

**Affiliations:** a Harvard School of Public Health, Department of Global Health and Population, 1633 Tremont Street, Boston, MA 02120, United States of America (USA).; b Muhimbili University of Health and Allied Sciences, Dar es Salaam, United Republic of Tanzania.; c Imperial College London, London, England.; d Harvard Medical School, Boston, USA.

## Abstract

**Objective:**

To determine the impact of intimate partner violence against women on children’s growth and nutritional status in low- and middle-income countries.

**Methods:**

We pooled records from 42 demographic and health surveys in 29 countries. Data on maternal lifetime exposure to physical or sexual violence by an intimate partner, socioeconomic and demographic characteristics were collected. We used logistic regression models to determine the association between intimate partner violence and child stunting and wasting.

**Findings:**

Prior exposure to intimate partner violence was reported by 69 652 (34.1%) of the 204 159 ever-married women included in our analysis. After adjusting for a range of characteristics, stunting in children was found to be positively associated with maternal lifetime exposure to only physical (adjusted odds ratio, aOR: 1.11; 95% confidence interval, CI: 1.09–1.14) or sexual intimate partner violence (aOR: 1.09; 95% CI: 1.05–1.13) and to both forms of such violence (aOR: 1.10; 95% CI: 1.05–1.14). The associations between stunting and intimate partner violence were stronger in urban areas than in rural ones, for mothers who had low levels of education than for women with higher levels of education, and in middle-income countries than in low-income countries. We also found a small negative association between wasting and intimate partner violence (aOR: 0.94; 95%CI: 0.90–0.98).

**Conclusion:**

Intimate partner violence against women remains common in low- and middle-income countries and is highly detrimental to women and to the growth of the affected women’s children. Policy and programme efforts are needed to reduce the prevalence and impact of such violence.

## Résumé

**Objectif:**

Déterminer l'impact de la violence conjugale envers les femmes sur la croissance et l'état nutritionnel des enfants dans les pays à revenu faible et intermédiaire.

**Méthodes:**

Nous avons regroupé les notes de 42 enquêtes démographiques et sanitaires menées dans 29 pays. Nous avons recueilli des données sur l'exposition des mères à des violences physiques ou sexuelles de la part du conjoint ainsi que sur les caractéristiques socioéconomiques et démographiques. Nous avons utilisé des modèles de régression logistique pour déterminer l'association entre violence conjugale et retard de croissance ou émaciation des enfants.

**Résultats:**

Une exposition à des violences conjugales a été signalée par 69 652 (34,1%) des 204 159 femmes ayant déjà été mariées qui étaient incluses dans notre analyse. Après ajustement en fonction d'un ensemble de caractéristiques, nous avons observé une association positive entre le retard de croissance chez l'enfant et l'exposition des mères à des violences conjugales de nature physique (rapport des cotes ajusté (RCa): 1,11; intervalle de confiance (IC) de 95%: 1,09–1,14), sexuelle (RCa: 1,09; IC 95%: 1,05–1,13) ou à ces deux formes de violence (RCa: 1,10; IC 95%: 1,05-1,14). L'association entre retard de croissance et violence conjugale était plus marquée en zone urbaine qu'en zone rurale; elle l'était également davantage pour les mères ayant un faible niveau d'éducation que pour celles ayant un niveau d'éducation plus élevé et apparaissait aussi plus importante dans les pays à revenu intermédiaire que dans ceux à revenu faible. Nous avons également observé une légère association négative entre l'émaciation et la violence conjugale (RCa: 0,94; IC 95%: 0,90-0,98).

**Conclusion:**

La violence conjugale exercée contre les femmes reste courante dans les pays à revenu faible et intermédiaire, au grand détriment des femmes et de la croissance de leurs enfants. Des efforts sont nécessaires en matière de politiques et de programmes afin de réduire la prévalence et l'impact de cette forme de violence.

## Resumen

**Objetivo:**

Determinar el impacto de la violencia de pareja contra las mujeres en el crecimiento y el nivel de nutrición infantil en países con ingresos bajos y medios.

**Métodos:**

Se recopilaron los resultados de 42 encuestas de demografía y salud en 29 países. Se recogieron datos sobre las características socioeconómicas y demográficas en cuanto a la exposición constante de una madre a violencia física o sexual por parte de su pareja. Se utilizaron modelos de regresión logística para determinar la relación entre la violencia de pareja y el retraso y la insuficiencia del crecimiento infantil.

**Resultados:**

Se registró exposición previa a violencia de pareja en 69 652 (34,1%) de las 204 159 mujeres casadas incluidas en nuestro análisis. Tras fijar una serie de características, se observó que el retraso del crecimiento infantil estaba relacionado con la exposición constante de la madre solamente a violencia de pareja física (coeficiente de posibilidades ajustado, CPa: 1,11; intervalo de confianza, IC, del 95%: 1,09–1,14) o solamente a violencia de pareja sexual (CPa: 1,09; IC del 95%: 1,05–1,13) y a ambas formas de violencia (CPa: 1,10; IC del 95%: 1,05–1,14). La relación entre la deficiencia del crecimiento y la violencia de pareja era mayor en zonas urbanas que en rurales, en madres con niveles bajos de educación que en mujeres con niveles altos de educación, y en países con ingresos medios que en países con ingresos bajos. También se observó una pequeña relación negativa entre la insuficiencia del crecimiento y la violencia de pareja (CPa: 0,94; IC del 95%: 0,90–0,98).

**Conclusión:**

La violencia de pareja contra las mujeres sigue siendo un problema habitual en países con ingresos bajos y medios y es altamente perjudicial para las mujeres y para el crecimiento de los hijos de las mujeres afectadas. Es necesario que las políticas y los programas se esfuercen por reducir la frecuencia y el impacto de este tipo de violencia.

## ملخص

**الغرض:**

تحديد أثر العنف الموجه ضد النساء من جانب شريك الحياة الحميم على نمو الأطفال والحالة التغذوية لديهم في البلدان منخفضة الدخل والبلدان متوسطة الدخل.

**الطريقة:**

عملنا على الاستفادة من المعلومات الواردة بسجلات مستمدة من استطلاعات للرأي تم إجراؤها في 29 دولة وبلغ عددها 42 استطلاعًا يرتبط بالخصائص السكانية والجوانب الصحية. وتم جمع البيانات المتعلقة بتعرض الأمهات على مدى حياتهن للعنف البدني أو الجنسي الذي يمارسه شريك الحياة الحميم، وكذلك البيانات المتعلقة بالعوامل الاجتماعية الاقتصادية والخصائص السكانية. واستخدمنا نماذج التحوف اللوجيستي لتحديد الارتباط بين العنف الممارس من جانب شريك الحياة الحميم ومعاناة الأطفال من التقزم والهزال.

**النتائج:**

إن النساء اللاتي شملهن التحليل الذي أجريناه ممن سبق لهن الزواج والبالغ عددهن 69,652 (بنسبة 34.1‏%) من إجمالي 204,159 امرأة أوردن معلومات تفيد بالتعرض فيما سبق للعنف الممارس من جانب شريك الحياة الحميم. وبعد إجراء التعديل بحسب مجموعة من الخصائص، تم التوصل إلى وجود ارتباط إيجابي بين التقزم لدى الأطفال وما تتعرض له الأمهات على يد شريك الحياة الحميم على مدى حياتهن من عنف بدني فقط (بنسبة احتمال معدَّلة بلغت: 1.11؛ بنسبة أرجحية مقدارها 95‏%: 1.09 – 1.14) أو عنف جنسي فقط (بنسبة احتمال معدَّلة بلغت: 1.09؛ ونسبة أرجحية مقدارها 95‏%: 1.05 – 1.13) وما تتعرضن له من كلا النوعين من ذلك العنف (بنسبة احتمال معدَّلة بلغت: 1.10؛ ونسبة أرجحية مقدارها 95‏%: 1.05–1.14). وظهر الارتباط بين التقزم والعنف الممارس من جانب شريك الحياة الحميم بصورة أكبر في المناطق الحضرية مقارنةً بالمناطق الريفية، وفي حالة الأمهات اللاتي حصلن على مستوى منخفض من التعليم مقارنةً بالنساء الحاصلات على مستوى أعلى من التعليم، وفي البلدان المتوسطة الدخل مقارنةً بالبلدان المنخفضة الدخل. كما توصلنا إلى وجود ارتباط سلبي بسيط بين الهزال والعنف الممارس من جانب شريك الحياة الحميم (بنسبة احتمال معدَّلة بلغت: 0.94؛ بنسبة أرجحية مقدارها ‏‏95%: 0.90–0.98).

**الاستنتاج:**

ما زال العنف الممارس من جانب شريك الحياة الحميم شائعًا في البلدان منخفضة الدخل والبلدان متوسطة الدخل حيث يتسبب في أضرار بالغة تلحق بالنساء وتؤثر على النمو لدى أولاد النساء المتضررات. وتقتضي الحاجة بذل الجهود المرتبطة بوضع السياسات والبرامج للحد من انتشار ذلك العنف وآثاره الضارة.

## 摘要

**目的:**

旨在确定低收入和中等收入国家中亲密伴侣暴力侵害妇女行为对儿童生长发育和营养状况的影响。

**方法:**

我们在 29 个国家搜集了 42 份人口统计与健康调查记录， 收集到与母亲在生活中遭受亲密伴侣的身体暴力和性暴力、社会经济和人口统计特征方面的数据。 我们使用逻辑回归模型来确定亲密伴侣暴力与儿童发育迟缓和消瘦之间的关联性。

**结果:**

在我们分析的 204 159 位已婚女性中，报告之前遭受过亲密伴侣暴力的人有 69 652 (34.1%) 位。 在对一系列特征进行调整后，我们发现儿童发育迟缓与以下因素有很强的关联性，即：母亲在生活中仅遭受身体暴力（调整后的优势比，aOR: 1.11；95% 置信区间，CI: 1.09–1.14）或者仅亲密伴侣性暴力 (aOR: 1.09; 95% CI: 1.05–1.13) 以及同时遭受这两种暴力 (aOR: 1.10; 95% CI: 1.05–1.14)。 对于发育迟缓与亲密伴侣暴力间的关联性，城市地区比农村地区关联性强、在教育水平低的母亲间比在教育水平更高的女性间的关联性强、中等收入国家比低收入国家关联性强。 我们还发现消瘦与亲密伴侣暴力之间的微小关联性 (aOR: 0.94; 95% CI: 0.90–0.98)。

**结论:**

对女性实施亲密伴侣暴力在低收入和中等收入国家比较普遍，同时对女性和受影响女性的孩子的成长非常不利。 需要开展政策和计划工作，以降低此类暴力的发生率和影响。

## Резюме

**Цель:**

Определить влияние насилия со стороны интимного партнера в отношении женщин на рост и качество питания детей в странах с низким и средним уровнем доходов.

**Методы:**

Были объединены документы 42 демографических опросов и обследований состояния здоровья населения, проведенных в 29 странах. Были собраны данные по подверженности матерей физическому и сексуальному насилию со стороны интимного партнера на протяжении жизни, а также социально-экономическим и демографическим характеристикам. С помощью моделей логистической регрессии была определена связь между насилием со стороны интимного партнера родителя и задержкой в росте и истощением детей.

**Результаты:**

О предыдущих случаях насилия со стороны интимного партнера сообщили 69 652 (34,1%) из 204 159 женщин, когда-либо состоявших в браке и включенных в анализ. После внесения поправки на ряд характеристик была обнаружена положительная связь между задержкой в росте детей и подверженностью матерей на протяжении своей жизни только физическому (скорректированное отношение шансов, СОШ: 1,11; 95%-й доверительный интервал, ДИ: 1,09–1,14) или сексуальному насилию со стороны интимного партнера (СОШ: 1,09; 95%-й ДИ: 1,05–1,13) и обеим формам такого насилия (СОШ: 1,10; 95%-й ДИ: 1,05–1,14). Связь между отставанием в росте и насилием со стороны интимного партнера была сильнее выражена в городской местности, чем в сельской, среди матерей с низким уровнем образования, чем среди женщин с высоким уровнем образования, и в странах со средним уровнем доходов, чем в странах с низким уровнем доходов. Также была выявлена незначительная отрицательная связь между исхуданием и насилием со стороны интимного партнера родителя (СОШ: 0,94; 95%-й ДИ: 0,90–0,98).

**Вывод:**

Насилие со стороны интимных партнеров в отношении женщин по-прежнему распространено в странах с низким и средним уровнем доходов и оказывает значительное негативное влияние на женщин и на рост детей подвергшихся насилию женщин. Необходимо разработать комплекс мер и составить программу действий, нацеленных на уменьшение распространенности и влияния такого насилия.

## Introduction

Intimate partner violence constitutes a major threat to the health and rights of women globally.^1^ According to a 2013 multi-country study by the World Health Organization (WHO), almost one third of all women have experienced physical and/or sexual violence by an intimate partner.^1^ A similar global estimate (30%) of the frequency of lifetime exposure to intimate partner violence among women was obtained in a meta-analysis based on 141 studies conducted in 81 countries.^2^ The study also showed that the frequency of such exposure was relatively high in central sub-Saharan Africa (65.6%), western sub-Saharan Africa (41.8%) and South Asia (41.7%).^2^

It seems likely that intimate partner violence against women has an impact on the growth and nutritional status of the children of the affected women. Some of the estimated 170 million children in low- and middle-income countries who are stunted^3^ may be suffering from the indirect effects of such violence. There have been several attempts to investigate possible links between intimate partner violence and stunting and wasting.^4^^–^^6^ In Liberia, children whose mothers had been exposed to sexual intimate partner violence were found to have relatively low mean weight-for-height *z*-scores and to be 2.6-fold more likely to be stunted than the other children in the study.^4^ Similarly, in a community-based study in Nicaragua, children of mothers who reported suffering intimate partner violence during pregnancy had relatively low height-for-age *z*-scores.^5^ A study with a nationally representative sample of children in India showed that, compared with the other children they investigated , the children of women who had been exposed to intimate partner violence in the previous year were 25% more likely to be stunted.^6^

There are several potential mechanisms through which intimate partner violence against women can have an effect on child growth and nutritional status. For example, such violence may increase the risk of – or, at least, share some contributing factors with – child abuse and neglect within the household. If observed by the children, such violence can cause childhood stress that, in turn, can decrease metabolic rates, physical growth and cognitive functioning.^7^ The partners of women in an abusive relationship may stop the women going to health clinics when their children are sick, stop the women paying for the health care of their children or severely limit the amount that the women can spend on food for their households.^8^^–^^10^ Intimate partner violence against a woman can have a negative impact on the woman’s physical and mental health, partly by limiting her access to health care for herself, including her access to antenatal care and skilled birth attendants.^11^^–^^14^ As it can cause maternal depression – which, in turn, can affect a woman’s ability to care for her child – such violence may contribute to childhood malnutrition even in households that have adequate food.^14^^–^^17^ Researchers have proposed conceptual frameworks that link direct and indirect pathways of intimate partner violence against women with child malnutrition through multidisciplinary literature review and data quantification.^18^^,^^19^

Most of the relevant data on the association between intimate partner violence and child growth and nutritional status have come from single-country studies with small samples that have given disparate results.^4^^,^^17^^,^^20^^–^^24^ A study using data collected in demographic and health surveys (DHS) in five countries to assess the relationship between intimate partner violence and stunting found that the strength of the relationship varied with the country involved.^18^ We therefore decided to evaluate the overall relationship between intimate partner violence against women and child growth and nutritional status for 29 low- and middle-income countries for which DHS data are publicly available.

## Methods

### Study population and design

We based our analysis on data collected from the DHS programme – i.e. nationally representative household surveys used to collect information on population-based indicators of health and nutrition across resource-poor countries.^25^

We combined data from the domestic violence module of the DHS with data collected in the women’s questionnaire. The 32-question domestic violence module, which was developed to measure the prevalence and consequences of physical and sexual violence, combines single threshold questions regarding prior experiences of intimate partner violence with a modified conflict tactics scale designed to measure spousal violence. Parts of the domestic violence module are designed to investigate non-spousal violence and intimate partner violence during pregnancy.^26^

**Fig. 1:**
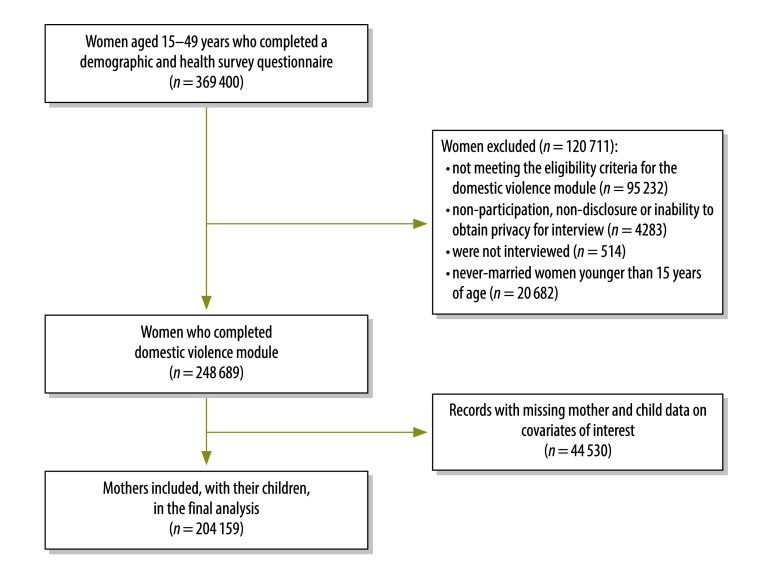
Flow diagram of the sample selection for the analysis on the association between intimate partner violence and child growth, 1998–2012

We used nationally representative data from 42 DHS conducted in a total of 29 countries (Table 1). We included all of the publicly available data from the standard DHS programme surveys and domestic violence modules completed between 1998 and 2012. In a typical DHS, all of the women aged 15–49 years living in a randomly selected set of households are interviewed. The domestic violence module is usually completed by just one –randomly selected – woman per surveyed household. Although 369 400 records were available from women who completed a demographic and health survey women’s questionnaire, 95 232 of the records did not meet eligibility criteria for the domestic violence module and 4283 had to be excluded because the selected interviewee refused to participate, the interview could not be conducted in private or the selected interviewee failed to be interviewed for another reason. Another 514 were not interviewed and were not included. We also excluded the 20 682 records that related to never-married women and interviewees younger than 15 years because, in most of our focus countries, only ever-married women older than 15 years were considered to be eligible to be interviewed about domestic violence. A further 44 530 records were incomplete and lacked data on at least one of our covariates of interest. We therefore confined our analysis to the records for 204 159 women and their children (Fig. 1).

**Table 1:** Demographic and health surveys included in the study on the association between intimate partner violence and child growth, 1998–2012

Country	Survey year	No. of women with completed domestic violence module
Azerbaijan	2006	1 669
Bangladesh	2007	2 474
Bolivia (Plurinational State of)	2003	9 093
Bolivia (Plurinational State of)	2008	6 359
Burkina Faso	2010	4 986
Cambodia	2000	1 780
Cameroon	2004	3 644
Colombia	2000	4 074
Colombia	2005	12 015
Colombia	2010	15 035
Dominican Republic	1999	4 346
Dominican Republic	2007	4 153
Gabon	2012	2 209
Ghana	2008	1 416
Haiti	2000	2 213
Haiti	2005	2 045
Haiti	2012	3 072
Honduras	2005	9 757
Honduras	2011	7 923
India	2005	37 387
Kenya	2003	4 103
Kenya	2008	4 467
Liberia	2007	3 351
Malawi	2004	8 372
Malawi	2010	4 486
Mali	2006	8 894
Mozambique	2011	5 226
Nepal	2011	1 888
Nigeria	2008	18 372
Peru	2000	11 829
Peru	2007	9 123
Republic of Moldova	2005	1 336
Rwanda	2005	3 178
Rwanda	2010	3 412
Sao Tome and Principe	1998	1 477
Timor-Leste	2009	2 467
Uganda	2006	2
United Republic of Tanzania	2010	5 459
Zambia	2001	3 998
Zambia	2007	4 449
Zimbabwe	2005	3 481
Zimbabwe	2010	3 669

### Intimate partner violence

For the DHS we investigated, physical violence was defined as the intentional use of physical force with the potential to cause injury or harm. Sexual violence was classified as any experience of unwanted or forced sexual activity. Self-reported maternal lifetime exposure to intimate partner violence was separated into four categories: (i) any; (ii) physical only; (iii) sexual only; and (iv) both physical and sexual.

### Child nutritional outcomes

As full maternal and child-level variables were only available for each interviewee’s last birth, we only investigated stunting as an indicator of linear growth and wasting as a measure of acute malnutrition in the youngest child of each interviewee. We calculated height-for-age and weight-for-height *z*-scores using the height and weight data from the DHS questionnaires and Anthro version 3.2.2 (WHO, Geneva, Switzerland).^27^^,^^28^ Stunting was defined as a height-for-age *z*-score that was less than minus two standard deviations from the median height-for-age given in WHO’s global database on child growth and malnutrition^29^ and wasting was defined as a weight-for-height *z*-score that was less than minus two standard deviations from the median weight-for-height given in the same database.^27^ Records giving *z*-scores that were lower than minus six or higher than six were assumed to be inaccurate and excluded from the analysis.

### Covariates

Based on previous studies,^18^^,^^21^^,^^24^ we included the following socioeconomic and demographic characteristics as covariates in the primary model: maternal age, employment status, level of education, marital status, partner’s level of education, rural/urban residence, use of contraception and wealth quintile, the number of children younger than five years in the household and the child’s age.

### Statistical analysis

We pooled all available observations for our analysis. Descriptive statistics were calculated for maternal- and child-level socioeconomic and demographic characteristics, both for the overall study sample and for each category of exposure to intimate partner violence. Unadjusted and adjusted logistic regressions were performed separately for each category of exposure to intimate partner violence. In the logistic regression models, each observation corresponded to a child, the main independent variable of interest being the child’s mother’s status of exposure to intimate partner violence. To adjust for the complex survey design used in the DHS, all standard errors were clustered at the level of the primary sampling unit.^30^^,^^31^ To control for unobservable differences in country-specific factors as well as differences in measurement, we included survey fixed effects in all of the regression models. To evaluate the significance of stratified associations, we used a pooled *ordinary least squares* model with intimate partner violence covariate interaction terms. All of the statistical analyses were conducted using Stata version 13 (StataCorp. LP, College Station, United States of America).

## Results

Table 2 shows descriptive statistics for the pooled, unweighted sample – i.e. respondents who completed the domestic violence module – and also for the full information sample used in our analysis. The mean age of the ever-married mothers was 28.4 years at the time of interview. In the final sample, about two thirds (130 031/204 159) of the households were in rural areas and 25% (52 440/204 159) of the interviewed mothers had never attended school. All of the children we investigated were aged 0–59 months.

**Table 2:** Descriptive characteristics of interviewees included in the study on the association between intimate partner violence and child growth, 1998–2012

Characteristic	No. of interviewees who completed domestic violence module (% of total)	No. of interviewees included in final analysis (% of those with characteristic)			*P^a^*
		Subgroup total	Reporting exposure to IPV	Reporting no exposure to IPV	
**Maternal**					
Age in years (*n* = 248 689)					
15–24	77 503 (31.2)	63 206	21 809 (34.5)	41 397 (65.5)	< 0.001
25–36	138 817 (55.8)	114 949	39 185 (34.1)	75 764 (65.9)	
37–49	32 369 (13.0)	26 004	8 658 (33.3)	17 346 (66.7)	
Residence (*n* = 248 689)					
Urban	93 341 (37.5)	74 128	25 994 (35.1)	48 134 (64.9)	< 0.001
Rural	155 348 (62.5)	130 031	43 658 (33.6)	86 373 (66.4)	
Wealth quintile (*n* = 248 689)					
Poorest	64 966 (26.1)	53 419	19 285 (36.1)	34 134 (63.9)	< 0.001
Poor	57 278 (23.0)	47 201	17 116 (36.3)	30 085 (63.7)	
Middle	50 667 (20.4)	41 674	14 906 (35.8)	26 768 (64.2)	
Richer	42 967 (17.3)	35 298	11 517 (32.6)	23 781 (67.4)	
Richest	32 811 (13.2)	26 567	6 828 (25.7)	19 739 (74.3)	
Education level (*n* = 248 684)					
None	62 079 (25.0)	52 440	16 850 (32.1)	35 590 (67.9)	< 0.001
Primary	100 024 (40.2)	79 937	29 556 (37.0)	50 381 (63.0)	
Secondary	70 422 (28.3)	59 033	20 264 (34.3)	38 769 (65.7)	
Higher	16 159 (6.5)	12 749	2 982 (23.4)	9 767 (76.6)	
Partner’s education level (*n* = 244 820)					
None	45 989 (18.8)	40 115	12 237 (30.5)	27 878 (69.5)	< 0.001
Primary	92 622 (37.8)	76 177	27 471 (36.1)	48 706 (63.9)	
Secondary	84 655 (34.6)	70 853	25 647 (36.2)	45 206 (63.8)	
Higher	21 554 (8.8)	17 014	4 297 (25.3)	12 717 (74.7)	
Employment status (*n* = 248 240)					
Employed	121 065 (48.8)	98 935	36 638 (37.0)	62 297 (63.0)	< 0.001
Unemployed	127 175 (51.2)	105 224	33 014 (31.4)	72 210 (68.6)	
Current marital status (*n* = 248 689)					
Married	166 546 (67.0)	136 984	43 740 (31.9)	93 244 (68.1)	< 0.001
Living with partner	64 323 (25.9)	52 857	18 808 (35.6)	34 049 (64.4)	
Widowed, divorced or separated	17 820 (7.2)	14 318	7 104 (49.6)	7 214 (50.4)	
Maternal height in cm (*n* = 234 936)					
< 150.0	55 404 (23.6)	44 171	15 770 (35.7)	28 401 (64.3)	< 0.001
150.0–190.0	179 476 (76.4)	151 160	51 763 (34.2)	99 397 (65.8)	
> 190.0	56 (0.0)	45	17 (37.8)	28 (62.2)	
Maternal body mass index (*n* = 234 695)					
< 18	18 217 (7.8)	16 029	6 164 (38.5)	9 865 (61.5)	< 0.001
18–30	199 426 (85.0)	165 751	56 987 (34.4)	108 764 (65.6)	
> 30	17 052 (7.3)	13 051	4 362 (32.3)	9 139 (67.7)	
**Child**					
Age in months (*n* = 248 689)					
0–23	101 418 (40.8)	81 237	26 440 (32.6)^b^	54 797 (67.5)^b^	< 0.001
24–59	147 271 (59.2)	122 922	43 212 (35.2)^b^	79 710 (64.9)^b^	
Sex (*n* = 248 689)					
Female	122 087 (49.1)	100 281	34 129 (34.0)^b^	66 152 (66.0)^b^	0.44
Male	126 602 (50.9)	103 878	35 523 (34.2)^b^	68 355 (65.8)^b^	

IPV: intimate partner violence.

^a^ Probability of observing statistically significant relationships between variables (Pearson’s *χ*-square). ^b^ The reported exposure to violence is that of the children’s mothers.

The overall prevalence of any lifetime exposure to intimate partner violence among the interviewed women was 34.1% (69 652). About one fifth (45 254) of the women claimed to have been slapped by their intimate partners and 16% (33 424) said that their intimate partners had pushed them, shaken them and/or thrown something at them. Nearly 9% (18 075) of the women said they had been punched by an intimate partner and 8% (16 298) said they had been physically forced into unwanted sex – including 2.5% (5513) who had been forced into first intercourse. While the prevalence of reported intimate partner violence decreased with increasing asset quintile as well as with increasing maternal and paternal education, such violence appeared common across all socioeconomic groups. Exposure to such violence was reported by more than 23% (2982) of the 12 749 interviewed mothers who were educated above secondary level and almost 26% (6828) of the 26 567 who belonged in the highest asset quintile. Of the 204 159 children in the sample, 29.6% (60 362) were stunted and 6.9% (14 184) were wasted.

Table 3 shows the unadjusted and adjusted associations between intimate partner violence, stunting and wasting. Overall, maternal exposure to any intimate partner violence increased the odds of stunting by 11% (adjusted odds ratio, aOR: 1.11; 95% CI: 1.09–1.14). Similar associations were found between stunting and maternal exposure to only the physical (aOR: 1.11; 95% CI: 1.09–1.14), only the sexual (aOR: 1.09; 95% CI: 1.05–1.13) or both forms of intimate partner violence (aOR: 1.10; 95% CI: 1.05–1.14). We also found small negative associations between wasting and both exposure to any intimate partner violence (aOR: 0.94; 95% CI: 0.90–0.98) and exposure only to the physical forms of such violence (aOR: 0.95; 95% CI: 0.91–0.99).

**Table 3:** Association between a woman’s exposure to intimate partner violence and stunting and wasting in her child, 29 countries, 1998–2012

Reported exposure	Stunting in child				Wasting in child			
	No.**^a^**	cOR (95% CI)	No.**^b^**	aOR^c^ (95% CI)	No.**^a^**	cOR (95% CI)	No.**^b^**	aOR^c^ (95% CI)
Any IPV	207 682	1.15 (1.12–1.17)	204 159	1.11 (1.09–1.14)	207 807	0.96 (0.92–1.00)	204 159	0.94 (0.90–0.98)
Physical IPV only	207 682	1.15 (1.12–1.18)	204 159	1.11 (1.09–1.14)	207 807	0.97 (0.93–1.01)	204 159	0.95 (0.91–0.99)
Sexual IPV only	187 758	1.11 (1.08–1.15)	184 350	1.09 (1.05–1.13)	187 882	1.03 (0.96–1.09)	184 350	1.00 (0.94–1.07)
Both physical and sexual IPV	202 613	1.14 (1.10–1.19)	199 128	1.10 (1.05–1.14)	202 738	1.07 (0.99–1.15)	199 128	1.04 (0.96–1.11)

aOR: adjusted odds ratio; CI: confidence interval; cOR: crude odds ratio; IPV: intimate partner violence.

^a^ Number of women included for the crude odds ratios.

^b^ Number of women included for the adjusted odds ratios.

^c^ Adjusted for maternal age, employment status, level of education, marital status, partner’s level of education, rural/urban residence, use of contraception and wealth quintile, the number of children aged less than five years in the household and the child’s age.

Table 4 shows the stratified results for stunting and wasting when using exposure to any intimate partner violence as the main independent variable of interest. For stunting, compared with the values for the other children in the sample, stronger positive associations with intimate partner violence were found among the children of women who had not been educated beyond primary level (aOR: 1.09; 95% CI: 1.07–1.12), who lived in urban areas (aOR: 1.22; 95% CI: 1.17–1.28), who lived in households in the two highest asset quintiles (aOR: 1.18; 95% CI: 1.14–1.22) and who lived in a middle-income country (aOR: 1.13; 95% CI: 1.10–1.17). The odds of child wasting were lower for the sampled children who were aged at least 24 months than for their younger counterparts (aOR: 0.88; 95% CI: 0.83–0.93).

**Table 4:** Stratified associations between a woman’s exposure to any intimate partner violence and stunting and wasting in her child, 29 countries, 1998–2012

Indicator	Stunting			Wasting		
	No.	aOR^a^ (95% CI)	*P*^b^	No.	aOR^a^ (95% CI)	*P*^b^
**Child age in months**						
< 24	81 237	1.11 (1.07–1.16)	0.35	81 237	1.01 (0.95–1.07)	< 0.05
≥ 24	122 922	1.11 (1.08–1.15)		122 922	0.88 (0.83–0.93)	
**Child sex**						
Female	100 281	1.10 (1.07–1.14)	0.28	100 281	0.92 (0.87–0.98)	0.38
Male	103 878	1.12 (1.09–1.16)		103 878	0.96 (0.91–1.02)	
**Residence**						
Rural	130 031	1.08 (1.05–1.11)	< 0.01	130 031	0.93 (0.88–0.98)	0.07
Urban	74 128	1.22 (1.17–1.28)		74 128	0.97 (0.90–1.06)	
**Household wealth quintile**						
Poorest, poor or middle	100 620	1.07 (1.03–1.10)	< 0.001	100 620	0.93 (0.87–0.98)	0.36
Richer or richest	103 539	1.18 (1.14–1.22)		103 539	0.96 (0.90–1.02)	
**Country**						
Low income	59 490	1.06 (1.02–1.10)	< 0.05	59 490	0.96 (0.89–1.03)	0.92
Middle income	144 669	1.13 (1.10–1.17)		144 669	0.92 (0.88–0.97)	
**Maternal education**						
None or primary	191 410	1.09 (1.07–1.12)	< 0.001	191 410	0.94 (0.90–0.98)	0.25
Secondary or higher	12 527	1.00 (0.86–1.17)		12 527	1.09 (0.84–1.43)	

aOR: adjusted odds ratio; CI: confidence interval.

^a^ Adjusted for maternal age, employment status, level of education, marital status, partner’s level of education, rural/urban residence, use of contraception and wealth quintile, the number of children aged less than five years in the household and the child’s age.

^b^ Based on a pooled *ordinary least squares* model with violence-category–covariate interaction terms.

## Discussion

As shown in previous studies,^1^^,^^2^ the results of our analysis highlight the high prevalence of intimate partner violence against women in low- and middle-income countries. They also indicate that maternal exposure to intimate partner violence substantially increases a child’s risk of stunting. A similar association has been observed before, in single-country studies in Bangladesh, Brazil, Haiti, India and Kenya.^17^^,^^32^^,^^21^^,^^22^^,^^21^ We were surprised to see that such exposure seemed to slightly reduce the risk of a child’s wasting but this result may be related to survivor bias in the context of a cross-sectional analysis. 

We found the association between intimate partner violence and child stunting to be relatively strong both for relatively rich women and for poorly educated women. In poorer households, the effects of maternal exposure to violence on child growth may be masked by the larger impacts of food insecurity,^33^ micronutrient deficiency^34^ and limited access to sanitation.^35^ The more educated women may carry more autonomy or relative agency within a relationship than their poorly educated counterparts – and thus be less vulnerable to the effects of intimate partner violence on their ability to care for their children.^36^^–^^40^

In some cases, maternal depression, which has been found to be associated with low birth weight,^41^ higher risk of prematurity^42^ and increased risk of obstetric complications,^43^ may be the link between violence experienced by a woman and her child’s poor growth. A meta-analysis showed that children whose mothers had depression were 1.4 times more likely to be stunted than the children of non-depressed mothers.^44^ Treatment of maternal depression may have benefits for the woman’s children in both the short-term – e.g. it may quickly give the woman sufficient energy to take her children to a clinic – and the long-term e.g. it may increase the woman’s self-efficacy and strengthen her autonomy in caring for her children.^36^^,^^37^^,^^40^

Our study has several limitations. First, the data set we used is cross-sectional. While it is possible that intimate partner violence was the result of child growth or malnutrition, such reverse causality seems relatively unlikely. Another disadvantage with the cross-sectional design is the potential for survivor bias, which may have resulted in the apparent increase in risk of wasting among children whose mothers had experienced intimate partner violence. The DHS were not designed to examine the associations in which we were interested. Although we controlled for several potentially confounding variables, there may have been confounding by other unknown factors. There may also have been residual confounding from the variables that were included in our multivariate analysis. For example, residual confounding may have occurred because of the challenges of measuring socioeconomic status in many different settings.

A further limitation of our study is our reliance on self-reported maternal exposure to intimate partner violence. Because of failures in recall and the effects of stigmatization, this approach is unlikely to capture overall exposure to such violence fully. Cultural attitudes towards, and the implications of, intimate partner violence in different countries may have contributed to underreporting. Further analysis, with longitudinal data, may provide insights on the temporal effects of exposure to such violence on our outcomes of interest. The study population we used, which was restricted to married women and did not include children who were living with relatives other than their mothers or in orphanages, may also have weakened the odds ratios that we calculated. Finally, the data we used did not allow us to control directly for child comorbidities – e.g. chronic diarrhoea and malaria – that might have been most common in areas with high levels of exposure to intimate partner violence prevalence and might have resulted in confounding bias.

Reductions in the burden of intimate partner violence against women are not only likely to improve the health and well-being of women but are also likely to improve the growth of many children. Although the programmatic reduction of intimate partner violence remains a challenge, there is emerging evidence, from randomized controlled trials, of several measures that can reduce such violence. For example, intimate partner violence appeared to be reduced by a programme of home visits in early childhood^45^ and by engaging women in microfinance and interventions against human immunodeficiency virus.^46^

The implications of the current study’s findings for policy are clear: given the high prevalence and detrimental impact of intimate partner violence against women, against both the women involved and their children, programmes to reduce such violence and attenuate its impact on maternal and child health are urgently needed. Initiatives to advance women’s autonomy, through access to education and economic opportunities, may offset the risk of intimate partner violence, potentially resulting in improvement in the growth and long-term development of many children.
